# Proliferating cell nuclear antigen restores the enzymatic activity of a DNA ligase I deficient in DNA binding

**DOI:** 10.1002/2211-5463.12209

**Published:** 2017-03-16

**Authors:** Carlos H. Trasviña‐Arenas, Cesar S. Cardona‐Felix, Elisa Azuara‐Liceaga, Corina Díaz‐Quezada, Luis G. Brieba

**Affiliations:** ^1^ Laboratorio Nacional de Genómica para la BiodiversidadCentro de Investigación y de Estudios AvanzadosIrapuatoGuanajuatoMéxico; ^2^ Posgrado en Ciencias GenómicasUniversidad Autónoma de la Ciudad de MéxicoMéxico; ^3^ Present address: Centro Interdisciplinario de Ciencias Marinas (CICIMAR‐IPN)Av. Instituto Politécnico Nacional. s/n.La PazBaja California Sur23096Mexico; ^4^ Present address: Cátedras CONACyTDirección Adjunta de Desarrollo CientíficoConsejo Nacional de Ciencia y TecnologíaAv. Insurgentes Sur 1582Ciudad de Mexico03940Mexico

**Keywords:** DNA ligase, PCNA, PIP box, protein–protein interaction

## Abstract

Proliferating cell nuclear antigen (PCNA) coordinates multienzymatic reactions by interacting with a variety of protein partners. Family I DNA ligases are multidomain proteins involved in sealing of DNA nicks during Okazaki fragment maturation and DNA repair. The interaction of DNA ligases with the interdomain connector loop (IDCL) of PCNA through its PCNA‐interacting peptide (PIP box) is well studied but the role of the interacting surface between both proteins is not well characterized. In this work, we used a minimal DNA ligase I and two N‐terminal deletions to establish that DNA binding and nick‐sealing stimulation of DNA ligase I by PCNA are not solely dependent on the PIP box–IDCL interaction. We found that a truncated DNA ligase I with a deleted PIP box is stimulated by PCNA. Furthermore, the activity of a DNA ligase defective in DNA binding is rescued upon PCNA addition. As the rate constants for single‐turnover ligation for the full‐length and truncated DNA ligases are not affected by PCNA, our data suggest that PCNA stimulation is achieved by increasing the affinity for nicked DNA substrate and not by increasing catalytic efficiency. Surprisingly C‐terminal mutants of PCNA are not able to stimulate nick‐sealing activity of *Entamoeba histolytica* DNA ligase I. Our data support the notion that the C‐terminal region of PCNA may be involved in promoting an allosteric transition in *E. histolytica* DNA ligase I from a spread‐shaped to a ring‐shaped structure. This study suggests that the ring‐shaped PCNA is a binding platform able to stabilize coevolved protein–protein interactions, in this case an interaction with DNA ligase I.

**Abbreviations**
DBD: DNA‐binding domain
DTT: dithiothreitol
ECL: enhanced chemiluminescence
EhDNAligI: *E. histolytica* DNA ligase I
EhPCNA: *E. histolytica* PCNA
HsDNAligI: human DNA ligase I
HsPCNA: human PCNA
IDCL: interdomain connector loop
PCNA: proliferating cellular nuclear antigen
PIP box: PCNA‐interacting peptide motif

Proliferating cell nuclear antigen (PCNA) encircles DNA while allowing its lateral movement and coordinates protein–protein interactions at macromolecular assemblies 1, 2. The homotrimeric structure of PCNA is conserved in human, crustacean, yeast, and protozoa 2, 3, 4, 5. Proteins such as translesion synthesis DNA polymerases, replicative DNA polymerases, or flap endonucleases interact with PCNA via an interaction motif dubbed PCNA‐interacting peptide motif (PIP box) 6, 7, 8. DNA ligase I is a multidomain protein involved in sealing DNA nicks at Okazaki fragments, and base (BER) and nucleotide (NER) excision DNA repair pathways 9, 10. DNA ligase I catalyzes the final step of those enzymatic processes 9, 11, 12. The role of DNA ligase I in a wide variety of DNA transactions is driven throughout its interaction with PCNA 13, 14. In contrast to other enzymes that bind to each of the three subunits of PCNA simultaneously, the interaction of DNA ligase I to PCNA obeys the stoichiometry of one ligase per PCNA trimer. The structure of human DNA ligase I (HsDNAligI) complexed with nicked DNA revealed that family I ligases consist of three domains: Adenylation, Oligonucleotide‐Oligosaccharide, and DNA binding 15. A structural model of HsDNAligI and human PCNA (HsPCNA) shows that their ring diameters of both proteins are of similar size and suggested a specific interaction surface between both proteins 15. Pascal and coworkers suggested that the initial interaction of DNA ligase with PCNA occurs via its PIP box and upon PCNA binding, DNA ligase I undergoes a rearrangement to a closed‐ring conformation 16. The DNA‐binding domain (DBD) of HsDNAligI interacts with PCNA suggesting that the interaction of DNA ligase and PCNA is not dependent on the PIP box 17 and electron microscopy studies support the notion that DNA ligase and PCNA interact via a conserved protein surface 18. Despite the existence of a surface interaction between HsPCNA and HsDNAligI, the effect of this interaction on the enzymatic activity of human DNA ligase is not as clear. A series of studies indicate that PCNA is unable to stimulate the nick‐sealing activity of DNA ligase I 19, 20. In contrast, other studies indicate that DNA ligases from *Homo sapiens* and *Sulfolobus sulfataricus* are stimulated by PCNA 16, 21.

Protozoan parasites like *Entamoeba histolytica* contain proteomes that are generally shorter than other eukaryotes 22, 23, 24. DNA ligase I of *E. histolytica* (EhDNAligI) is 233 residues shorter than DNA ligase I of *H. sapiens* 25. PCNA from *E. histolytica* (EhPCNA) presents a homotrimeric structure similar to HsPCNA 4. *Entamoeba histolytica* harbors functional glycoslyases and a family A DNA polymerase indicating the presence of a base excision repair pathway in this parasite in which EhDNAligI, as the solely DNA ligase in this parasite, may be involved 26, 27, 28. In order to shed light into the functional interactions of DNA ligase I with PCNA, we used EhDNAligI as a minimal model system. Herein, we show that PCNA stimulates the nick‐sealing activity of a PIP box‐deleted mutant and restores the enzymatic activity of a DNA ligase that is impaired DNA binding.

## Results

### EhDNAligI contains a minimal scaffold for DNA ligation

**Figure 1:**
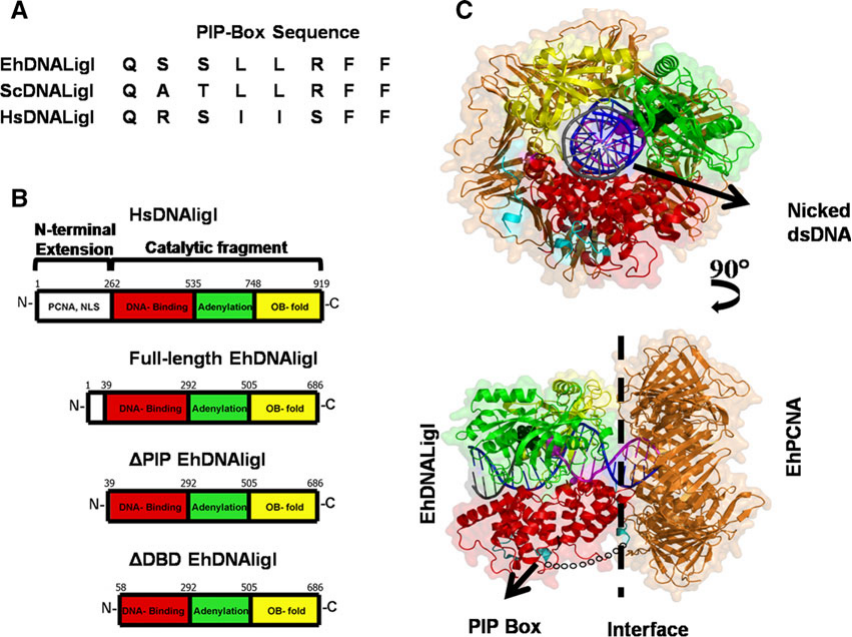
EhDNAligI is a minimal scaffold for DNA ligation. (A) Amino acid sequence alignment of PIP boxes from *H. sapiens* and *E. histolytica* in comparison to the consensus sequence. (B) Family I DNA ligases are multidomain proteins composed of a catalytic core and an N‐terminal region. Human DNA ligase consists of 919 amino acids; its catalytic core is divided into three domains: DBD (red), adenylation domain (green), and Oligonucleotide‐oligosaccharide‐binding domain (yellow). Its N‐terminal region consists of 262 amino acids and is involved in protein–protein interactions. HsDNAligI interacts with PCNA via an eight‐amino‐acid motif dubbed PIP box. EhDNAligI consists of 686 amino acids, contains a catalytic core of similar length to human DNA ligase with a reduced N‐terminal region of only 39 amino acids. This N‐terminal region EhDNAligI contains a consensus PIP box motif. ΔPIP lacks the first 39 amino acids in which the PIP box (residues 2–9) and a predicted disordered region are deleted. ΔDBD lacks the first 58 amino acids in which the PIP box, a predicted disordered region and two putative α‐helixes that are predicted to stabilize the DBD domain are deleted. (C) Structural model of EhDNAligI in complex with EhPCNA. The structural domains of EhDNAligI are colored accordingly to the cartoon representation. The upper panel shows in front the EhDNAligI and in the back EhPCNA, a nicked DNA molecule of 40 bp is in a ball‐stick representation. The lower panel is a 90° rotation in which the PIP box is colored blue. The 21 amino acids missing in the structural model of EhDNAligI are indicated by a broken chain. The interface between PCNA and EhDNAligI is signaled with an arrow.

**Figure 2:**
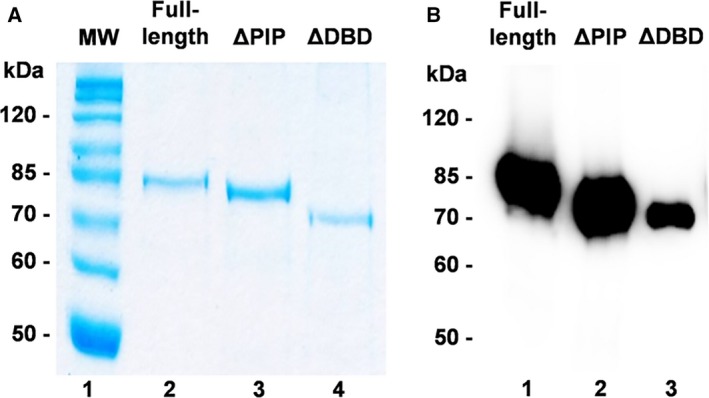
Protein purification and adenylation of full‐length and N‐terminal deletion mutants. (A) Purification of full‐length and N‐terminal deletion mutants. SDS/PAGE Coomassie blue stained; lane 1, Molecular weight markers; lane 2, full‐length; lane 3, ∆PIP; lane 4, ∆DBD. (B) Adenylation of full‐length and deletion mutants. About 1 μg of each purified protein was incubated with 1 μCi [α‐^32^P] ATP for 10 min at room temperature in the presence of 50 mm Tris/HCl (pH 7.5), 5 mm DTT, and 10 mm MgCl_2_.The samples were resolved by 10% SDS/PAGE and exposed to autoradiography.

EhDNAligI carries out all the three steps of a ligation reaction 25. An amino acid sequence alignment of the sequences of PIP boxes from *H. sapiens* and *E. histolytica* shows that both enzymes contain canonical PIP box sequences (Fig. 1A). The length of the catalytic fragment used to solve the crystal structure of HsDNAligI in complex with ds nicked DNA is similar to the length of full‐length EhDNAligI 15, 25. The main difference between these two DNA ligases resides in the length of their N‐terminal extensions; in *H. sapiens* this extension consists of 262 amino acids, whereas in *E. histolytica* the N‐terminal extension is of 39 amino acids (Fig. 1B) 25. At their N‐terminal extensions both DNA ligases contain a PCNA‐binding motif (PIB box) and a nuclear localization signal. However, human DNA ligase also contains a binding site for DNA polymerase β 29. The catalytic core of both DNA ligases shares 67% amino acid identity (Fig. S1). Based on the crystal structure of HsDNAligI and the structural alignment with EhDNAligI, we made a structural homology model of EhDNAligI and its interaction with EhPCNA (Fig. 1C). With basis on this alignment and in order to test the interaction of family I DNA ligases with PCNA, we constructed two EhDNAligI deletion mutants (Fig. 1B). To study the role of DNA ligase's PIP box in PCNA binding, we constructed EhDNAligI mutant that lacks its first 39 amino acids including the PIP box, located between residues 2 and 9 (hereinafter called ΔPIP) and a second deletion mutant that lacks the PIP box and two putative α‐helixes of the DBD (hereinafter called ΔDBD). As the deletion of these two α‐helixes would result in ligase with a defective DBD, we predicted that the ΔDBD construct would result in a DNA ligase with an impaired ability to bind a nicked DNA substrate. EhDNAligI and the two deletion mutants were purified to homogeneity as observed in a 10% SDS/PAGE Coomassie blue‐stained gel (Fig. 2A, lanes 2–4).

### Full‐length and deletion mutants can perform the first step in the nick‐sealing reaction

The first step in a ligation reaction is the adenylation of a conserved lysine by ATP to form a DNA ligase–AMP complex. During *in vitro* purification, heterologous‐expressed EhDNAligI is present mostly as a ligase–AMP complex 25. Thus, full‐length EhDNAligI and deletion mutants were deadenylated using molar excess of pyrophosphate. Complete deadenylation was corroborated by the lack of nick‐sealing activity in the absence of exogenous ATP (data not shown). The ability of these constructs to self‐adenylate was tested by incubating the purified proteins in the presence of [α‐^32^P]ATP (Fig. 2B, lanes 1–3). As observed, full‐length and ΔPIP exhibited similar degrees of adenylation. However, the extent of adenylation in ΔDBD is reduced (Fig. 2B, lane 3). This may indicate that the partial deletion in DBD alters the local fold of this construct, so the catalytic lysine is differentially exposed to react with ATP. The addition of PCNA in the absence of nicked DNA does not have an effect on the ratio of self‐adenylation by all EhDNAligI proteins (data not shown).

### ΔDBD is unable to bind at DNA substrate; however, PCNA can restore binding capability

**Figure 3:**
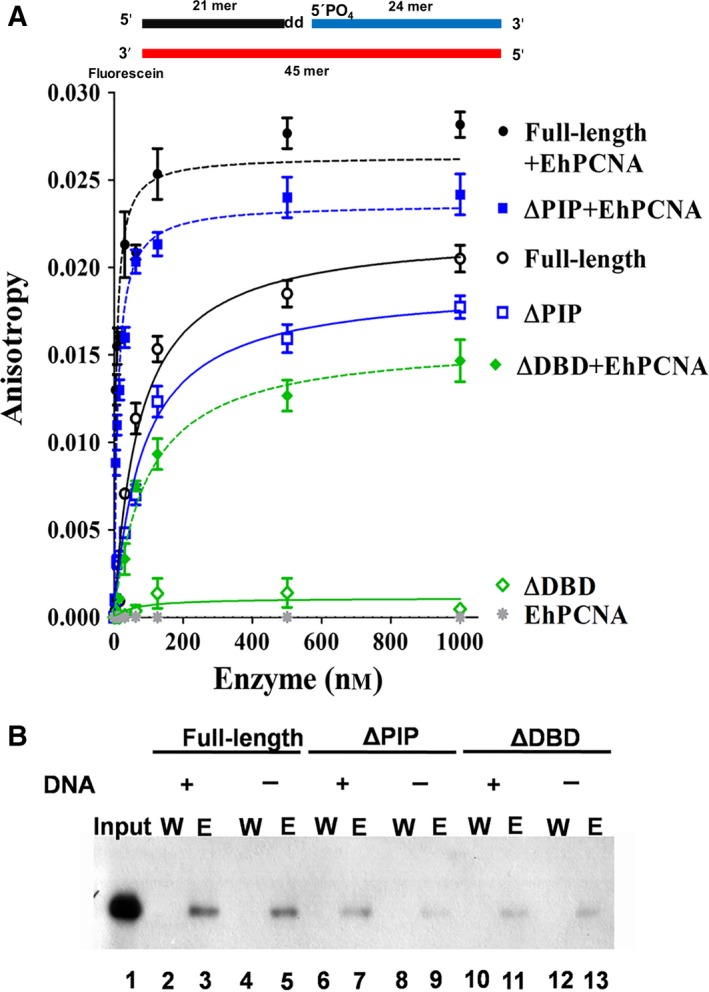
Effect of EhPCNA on DNA binding capability of full‐length and N‐terminal deletion mutants. (A) Enhancement and restoration of the DNA binding activity from full‐length and deletion mutants measured by anisotropy analysis. In the Y axis is plotted the anisotropy changes produced by increasing the concentration of enzyme. The lines are displayed in the following colors: full‐length black; ΔPIP, blue; and ΔDBD, green. Solid lines are anisotropy changes only in the presence of DNA ligase, while dotted lines plotted in the anisotropy changes in the presence of enzymes and EhPCNA. A control of binding from EhPCNA was included (gray line). Each point is the average of three independent experiments (B). Determination of the interaction between EhDNAligI with EhPCNA by pull‐down analysis. Purified (His)_6_ full‐length and deletion mutants were incubated with nickel‐chelating resin and EhPCNA as described in material and methods. Lane 1, 10% of input of EhPCNA; lanes 2 and 4, resin wash of full‐length in the presence and absence of substrate, respectively; lane 3 and 5, inmunodetection of EhPCNA bind at full‐length in the presence and absence of substrate, eluted with 500 mm imidazole; lanes 6 and 8, resin wash of ∆PIP; lanes 7 and 9, elution of ∆PIP; lanes 10 and 12, resin wash of ∆DBD; lanes 11 and 13; elution of ∆DBD. A diagram showing the fluorescein‐labeled double‐stranded nicked substrate is present in the upper part of the figure, the 3′ dideoxyCTP that halts DNA ligations is labeled with the legend.

The second step of a ligation reaction is the binding of DNA ligase to a 5′ PO_4_ nicked DNA. The DNA binding affinity of full‐length and truncated EhDNAligI was measured by anisotropy (Fig. 3A and Table 1). In the presence of EhPCNA, the titration of full‐length EhDNAligI revealed an 11‐fold increase in binding affinity compared with the value in the absence of EhPCNA, with dissociation constants of 6.2 nm and 72.1 nm, respectively. ΔPIP exhibited a *K*_d_ = 82.1 nm that significantly decreased in the presence of EhPCNA (*K*_d_ = 10.2 nm). Thus, indicating that the PIP box of EhDNAlig I is not necessary to establish an interaction with EhPCNA. However, the most interesting observation is the restoration of the capability of ΔDBD upon PCNA addition to bind to a 3′‐FAM‐labeled nicked DNA substrate. The affinity constant of ΔDBD in the presence of EhPCNA (*K*_d_ = 95.5 nm) is near to the observed value for full‐length and ΔPIP in the absence of EhPCNA. This observation is in agreement with previous suggestions that indicate that PCNA reorganizes the disrupted DNA‐binding domain of ΔDBD by an interaction that is not dependent of the PIP box 17. We were curious to know if the enhancement on DNA binding is specific for EhPCNA or if an orthologous PCNA, like HsPCNA, could enhance the binding of EhDNAligI to nicked DNA. HsPCNA increases the binding of full‐length EhDNAligI, as the *K*_d_ for nicked DNA varies from 72.1 to 11.35 nm (Fig. S2). However, the presence of HsPCNA on ΔPIP and ΔDBD has no detectable effect on nicked DNA binding (Fig. S2). We rationalize that the canonical PIP box of EhDNAligI interacts with the interdomain connector loop (IDCL) of HsPCNA and forms a stable complex mediated by the PIP box of the full‐length enzyme; however, in the cases of ΔPIP and ΔDBD, in which the PIP box is eliminated, no binding is observed because the ring‐shaped interaction surface of EhPCNA and HsPCNA is different 4. These data suggest that the initial interaction between DNA ligase and PCNA occurs via an interaction with the PIP box and that a second specific interaction between DNA ligase and PCNA occurs via a specific interface between both proteins. Thus, our results indicate that the initial binding of DNA ligase to trimeric PCNA occurs via its PIP box and suggest the presence of a surface interaction between EhDNAligI and EhPCNA.

**Table 1:** Kinetic parameters of EhDNAligI and its deletion mutants

Protein(s)	Steady state				Single turnover	DNA binding
	*K*_m_ (nm)	*V*_max_ (nm·min^−1^)	*k*_cat_ (min^−1^)	*k*_cat_/*K*_m_ (m ^−1^·s^−1^)	*k*_st_ (min^−1^)	*K*_d_ (nm)
Full‐length	68 ± 9.6	6.2 × 10^−3^ ± 1.59 × 10^−4^	2.2 ± 0.1	5.6 × 10^5^	2.1 ± 0.2	72.1 ± 9.3
Full‐length + EhPCNA	6.3 ± 1.1	1.08 × 10^−2^ ± 2.7 × 10^−4^	3.9 ± 0.1	10.4 × 10^6^	2.2 ± 0.1	6.2 ± 1.0
∆PIP	77.8 ± 13	5.6 × 10^−3^ ± 1.7 × 10^−4^	2 ± 0.1	4.4 × 10^5^	2 ± 0.09	82.1 ± 9.1
∆PIP + EhPCNA	6.3 ± 1.1	8.8 × 10^−3^ ± 2.2 × 10^−4^	3.2 ± 0.1	8.4 × 10^6^	2.3 ± 0.3	10.2 ± 1.1
∆DBD	–	–	–	–	–	–
∆DBD + EhPCNA	81.8 ± 12	5.08 × 10^−3^ ± 1.3 × 10^−4^	1.8 ± 0.08	3.7 × 10^5^	1.7 ± 0.3	95.5 ± 14.7
EhPCNA	–	–	–	–	–	–

Each experiment is the average of triplicate determinations.

### EhPCNA physically binds to full‐length and deletion mutants

To corroborate an EhDNAligI and EhPCNA interaction that is not solely dependent of the PIP box, we carried out a pull‐down assay using a nickel affinity column. Full‐length EhDNAligI and its deletion mutants were (His)_6_ tagged at their N terminus, whereas EhPCNA did not harbor a histidine tag. As DNA ligases adopt different conformations in apo and DNA bound forms 15, 16, we were curious to know if the addition of nicked DNA can influence the DNA ligI interaction with PCNA. We found that EhPCNA was not detectable by western blot analysis after extensive washing; however, western blotting of the eluate using imidazole in the elution buffer indicates the presence of EhPCNA indicating the formation of complex between His‐tagged EhDNAligI and EhPCNA (Fig. 3B, lanes 3 and 5). In the case of ΔPIP and ΔDBD constructs, the intensity of the western blot band decreases by less than half, in comparison to the band observed for the full‐length DNA ligase; this indicates that EhPCNA is able to remain bound to ΔPIP and ΔDBD constructs after an extensive wash (Fig. 3B, lanes 7, 9, 11 and 13). Thus, western blot analysis corroborate that both truncated DNA ligases are capable to interact with EhPCNA. These data support that DNA ligase I interacts with PCNA even in the absence of nicked DNA and that this interaction is independent of the PIP box. Our observations agrees with previous work that demonstrated that the DBD of human DNA ligase binds to HsPCNA 16, 17.

### EhPCNA but not HsPCNA rescues the nick‐sealing activity of a DNA‐binding‐defective EhDNAligI

**Figure 4:**
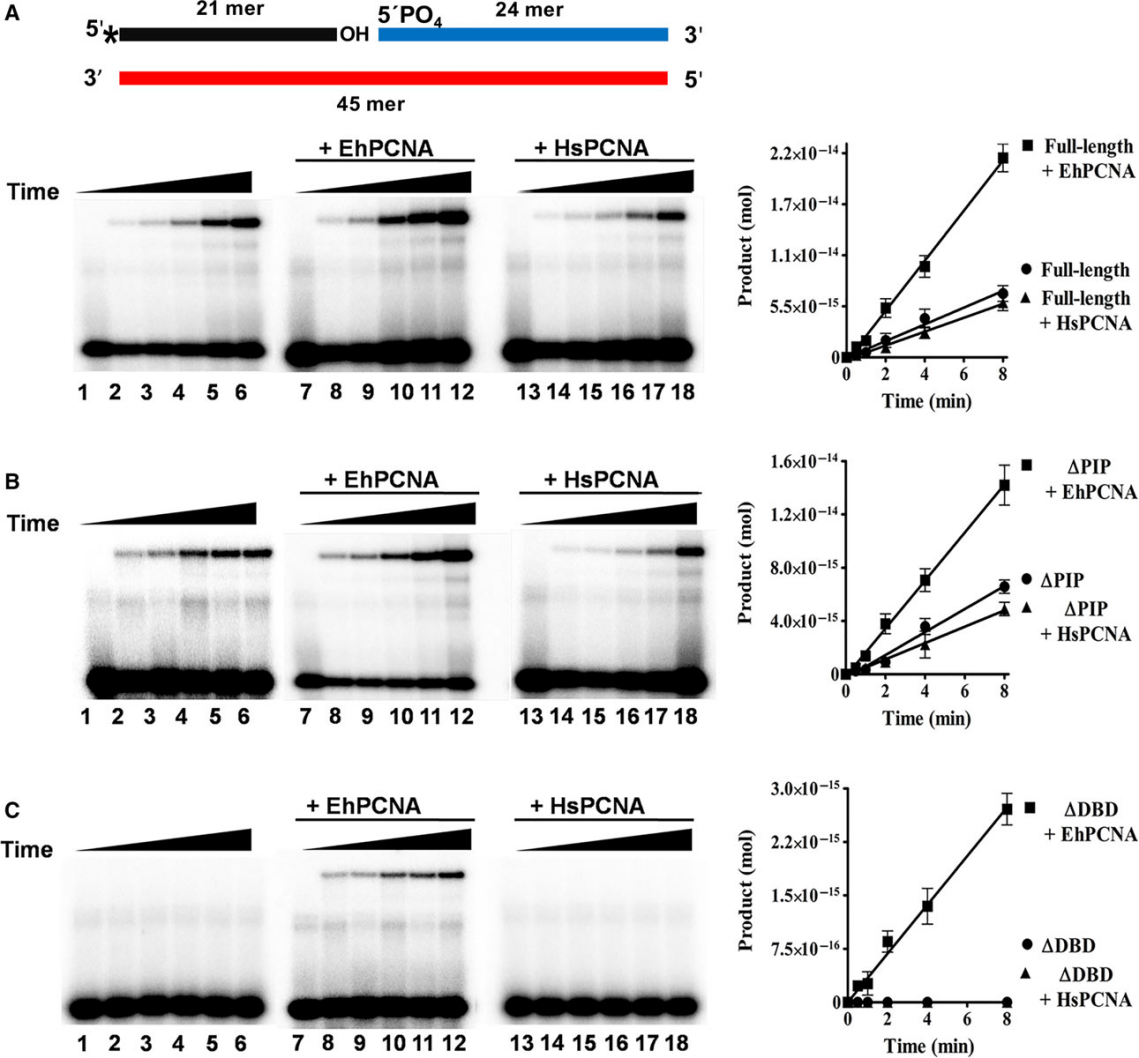
EhPCNA enhances the nick‐sealing activity of EhDNAligI and ΔPIP and restores the activity of ΔDBD. Reaction containing 500 fmol of [γ‐^32^P]‐labeled DNA substrate, 50 fmol of EhDNAligI and deletion mutants, and 150 fmol of EhPCNA or HsPCNA was incubated at room temperature for 0, 1, 2, 4, 8, and 16 min, aliquots were quenched at the indicated times adding stop buffer and loaded into a 15% polyacrylamide/8M urea gels. (A) Enzymatic activity of full‐length; lanes 1–6, analysis of time course activity only with full‐length; lanes 7–12, enzymatic activity when EhPCNA has been added; lanes 13–18, enzymatic activity with HsPCNA. (B and C) Time course activity with ∆PIP and ∆DBD deletion mutant as explained in A. Data are presented as pmol of ligated product; these values are plotted for EhDNAligI and deletion mutant as a function of incubation time. A diagram showing the double‐stranded nicked substrate is present in the upper part of the figure. The radioactive label at 5′ end of the 21 mer is indicated by an asterisk.

In order to understand the effect of EhPCNA on EhDNAligI nick‐sealing activity, we carried out a multiple enzyme turnover experiment. The activity for full‐length and deletion mutants was measured in a time course nick‐sealing reaction (Fig. 4A, lanes 1–6 and 4B, lanes 1–6, respectively). Data indicate that the deletion of the first 39 residues of EhDNAligI does not affect activity; in contrast, the ΔDBD mutant is not an active DNA ligase (Fig. 4C, lanes 1–6). No enzymatic activity is observed even with a 25‐fold molar increase of ΔDBD and a prolonged time course (Fig. S3). We tested the effect of the addition of sixfold molar excess of EhPCNA with respect to full‐length and truncated DNA ligases in product accumulation. EhPCNA stimulates the overall nick‐sealing reaction of full‐length and ΔPIP (Fig. 4A, lanes 7–12 and 4B, lanes 7–12). Moreover, we observe that EhPCNA reestablishes the enzymatic activity of the inactive ΔDBD ligase (Fig. 4C, lanes 7–12). The ring‐shaped structure of PCNA and the IDCL is conserved among PCNAs from *H. sapiens*,* Saccharomyces cerevisiae*, and *E. histolytica* 3, 4. As the role of PCNA in the catalytic enhancement of the nick‐sealing step by DNA ligases is controversial 16, 21, we tested if an orthologous PCNA from *H. sapiens* would influence the nick‐sealing activity of EhDNAligI. HsPCNA does not influence the nick‐sealing rate of full‐length (Fig. 4A, lanes 13–18) and ΔPIP EhDNAligases (Fig. 4B, lanes 13–18). The nick‐sealing activity of ΔDBD is not restored by HsPCNA (Fig. 4C, lanes 13–18). To test if the ΔDBD nick‐sealing activity was not restored because the decrease in binding affinity between EhDNAligI and HsPCNA, we systematically increased the concentration of HsPCNA. Even a 36‐fold molar excess HsPCNA is not able to restore the enzymatic activity of ΔDBD (data not show). As HsPCNA stimulates the binding of full‐length EhDNAligI at nicked DNA (Fig. S2), we suggest that the catalytic rate enhancement of EhPCNA to EhDNAligI may be due to a conformational change that occurs after the formation of the ternary PCNA–DNA ligase‐nicked DNA complex.

### Effect of EhPCNA on kinetic parameters of full‐length and deletion mutant

In order to understand the mechanism by which EhPCNA increases the activity of EhDNAligI, we measured kinetic parameters for ATP‐dependent ligation under excess of nicked DNA (Table 1). We obtained catalytic constants of *K*_m_ = 68 nm and a *k*_cat_ of 2.2 min^−1^ for full‐length EhDNAligI and similar values were obtained for ∆PIP (*K*_m_ = 77 nm and *k*_cat_ = 2 min^−1^). Likewise, similar catalytic efficiencies were observed for both enzymes (full‐length *k*_cat_/*K*_m_ = 5.6 × 10^5^ m ^−1^·s^−1^ and ΔPIP *k*_cat_/*K*_m_ = 4.4 × 10^5^ m ^−1^·s^−1^). As expected, these constants could not be calculated for the ∆DBD mutant in the absence of EhPCNA. In the experiments performed in the presence of EhPCNA, we observed a decrease of approximately 10‐fold in *K*_m_ and an increase of twofold in *k*_cat_ (*K*_m_ = 6.3 nm and *k*_cat_ = 3.9 min^−1^) for the full‐length DNA ligase. A similar phenomenon was observed for ΔPIP (*K*_m_ = 6.3 nm and *k*_cat_ = 3.2 min^−1^). Therefore, in both cases, EhPCNA produces an increase of at least 18‐fold in the catalytic efficiency with respect to the values in the absence of EhPCNA (full‐length *k*_cat_/*K*_m_ = 10.4 × 10^6^ m ^−1^·s^−1^ and ΔPIP *k*_cat_/*K*_m_ = 8.4 × 10^6^ m ^−1^·s^−1^).

The catalytic constants of ∆DBD in the presence of EhPCNA are similar to the values of full‐length DNA ligase without EhPCNA (*K*_m_ = 81.8 nm and *k*_cat_ = 1.8 min^−1^). This indicates that EhPCNA is able to stimulate (full‐length and ∆PIP) and restore (∆DBD) enzymatic activity. Similar effects of PCNA over catalytic constants has been reported for others enzymes like DNA polymerase δ 30, flap endonuclease 1 (FEN1) 31, and DNA polymerase κ 32.

### EhPCNA alters kinetic parameters for nick‐sealing but not single turnover

Single‐turnover kinetics allows the evaluation of rate constants on the formation of a specific catalytic step 33. In order to determine the effect of EhPCNA on the rate constant for single‐turnover ligation (*k*_st_), we determined the rate constants in the presence and absence of EhPCNA (Table 1). The *k*_st_ of the truncated human DNA ligase is ~ 40 min^−1^ 15, the measured value for full‐length EhDNAligI is *k*_st_ = 2.1 min^−1^ at 25 °C (Table 1). This value is 20 times lower than the value observed for human DNA ligase I; however, the rate constant is in the range of the observed values for other DNA ligases 34. From the single‐turnover kinetic analysis, we have two observations: first, the deletion of the first 39 amino acids (ΔPIP) does not have a significant effect in rate constant for single‐turnover ligation (full‐length *k*_st_ = 2.1 min^−1^ and ΔPIP *k*_st_ = 2 min^−1^) and second, EhPCNA is not able to significantly alter the rate constant for full‐length and ΔPIP DNA ligases (full‐length *k*_st_ = 2.2 min^−1^, ΔPIP *k*_st_ = 2.3 min^−1^). Interestingly, ΔDBD in the presence of EhPCNA exhibits a similar rate constant as that observed for full‐length and ΔPIP DNA ligases (*k*_st_ = 1.7 min^−1^). Our data indicate that as in the reported stimulation of HsPCNA to HsDNAligI, the EhPCNA stimulation to EhDNAligI is due to a increase of the binding of EhDNAligI to nicked DNA and favoring the turnover (substrate binding and product release, respectively) but not by altering step 3 (nick‐sealing) of the ligation reaction.

### Exonuclease footprinting indicates that EhPCNA binds to EhDNAligI and form a stable complex

**Figure 5:**
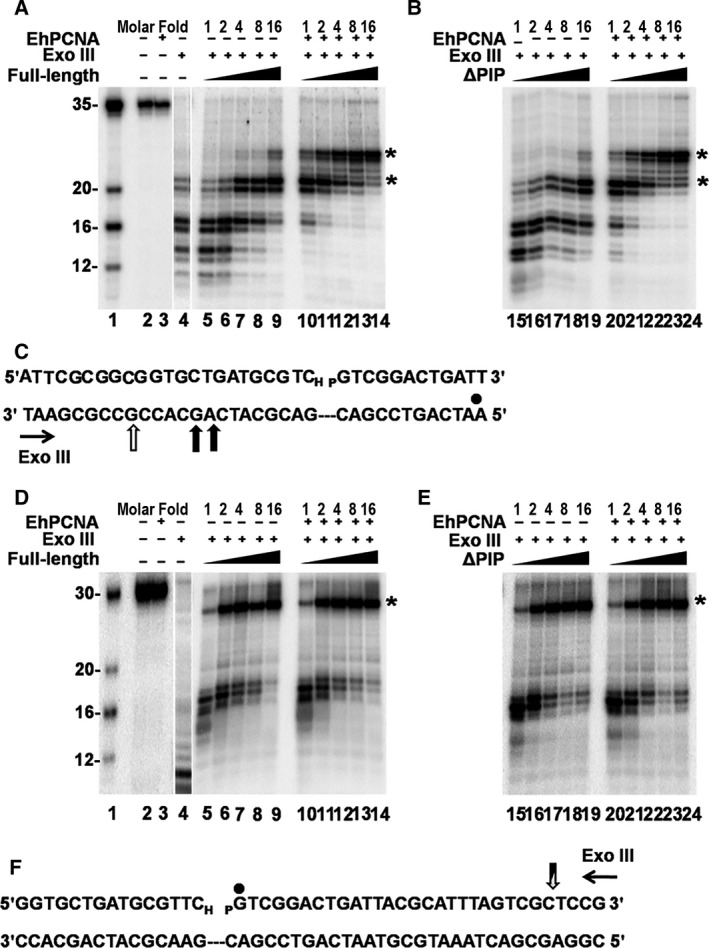
Footprinting of EhDNAligI–EhPCNA (A) 3′OH exonuclease III footprinting, 10 μL reaction mixture containing 100 fmol of substrate (lane 2) was incubated with 4 pmol of EhPCNA (lane 3) or only in the presence of exonuclease III (lane 4) as a degradation control. Increased concentrations of full‐length (lanes 5–9) and full‐length plus EhPCNA (lanes 10–14) were incubated to 20 °C for 8 min. (B) increased concentrations of ∆PIP (lanes 15–19) and ∆PIP plus EhPCNA (lanes 20–24) were incubated under the same condition. The molar fold of EhDNAlig or EhDNAlig–EhPCNA over labeled nicked DNA for each reaction is indicated. The asterisks indicate the formation of a well‐defined boundary. A set of four oligonucleotides (35, 20, 16, and 12 mer) was used as a ladder (lane 1). (C) Representation of the substrate to delimit the protection by EhDNAligI and EhPCNA by exonuclease III digestion. The radiolabeled strand is indicated by black dot. The vertical black arrows in the template strand depict the boundary of the protection of EhDNAligI, while the open arrows indicate the additional protection of EhPCNA in the same strand. (D) The footprinting reaction was performed under the same conditions as in panel A but with different substrate to determine the 5′PO_4_ exconuclease III footprinting. Increasing concentrations of EhDNAligI (lanes 5–9) and EhDNAligI and EhPCNA (lanes 10–14) were digested with exonuclease III. (E) Increased concentrations of ∆PIP (lanes 15–19) and ∆PIP in combination with EhPCNA (lanes 20–24) were tested under same conditions as in panel B. (F) Structure of the substrate used in footprinting reaction, black dot shows the labeled DNA downstream of the nick. A vertical arrow in the labeled oligonucleotide depicts the boundary of exonuclease footprinting, in this case the boundary is in the same position for EhDNAligI and EhPCNA.

In order to investigate if the absence of the PIP box alters the binding interface between EhPCNA and EhDNAligI, we determined the boundaries between binary (EhDNAligI–DNA) and ternary complexes (EhDNAligI–EhPCNA–DNA) with respect to nicked DNA by exonuclease III footprinting. As ΔDBD does not form stable complex on nicked DNA even in the presence of EhPCNA, we carried out exonuclease III footprinting assays using only full‐length and ΔPIP. We used two different substrates, first a 5′PO_4_ radiolabeled template oligonucleotide annealed to an upstream 23 mer in which the last nucleotide is a ddNMP to arrest the ligation reaction (Fig. 5C). This substrate was used to determine the boundary on the 3′OH. In second substrate, the 5′PO_4_ downstream strand on the DNA nick was radiolabeled (Fig. 5F). Footprinting reactions were carried out with increasing amounts of full‐length and ΔPIP with molar excess of EhPCNA.

Digestion with exonuclease III in the absence of EhDNAligI resulted in a nuclease‐resistant 16 mer (Fig. 5A, lane 4) that is digested to smaller products over time (data not shown). Increased concentrations of EhDNAligI resulted in the appearance a stable 21 mer. As the initial oligonucleotide consist of 35 nucleotides (Fig. 5A, lane 1), we concluded that the boundaries of EhDNAligI consist of 13–14 nucleotides 3′OH upstream of the nicked DNA (Fig. 5A, lanes 5–9). As expected, the addition of EhPCNA increases the protection of five extra nucleotides in the 3′OH (Fig. 5A, lanes 10–14). The exonuclease footprinting using ΔPIP shows the same footprint pattern as the full‐length ligase in the presence and absence of EhPCNA (Fig. 5B). Thus, the addition of EhPCNA changes the 3′OH footprinting by protecting five extra nucleotides. The presence of a sharp band indicates the formation of a stable complex.

The footprinting reaction at the 5′PO_4_ side of the nick without DNA ligase produces a band of nine nucleotides (Fig. 5D, lane 4) that is digested to approximately four nucleotides overtime (data not shown) and increasing concentrations of DNA ligase resulted in the appearance of a sharp band of 26 nucleotides (Fig. 5D, lanes 5–9). As expected, the addition of EhPCNA does not have any effect in the 5′PO_4_ boundary (Fig. 5D, lanes 10–14). Our exonuclease footprinting with ΔPIP displayed the same footprinting pattern as full‐length ligase in the presence and absence of EhPCNA (Fig. 5E). The exonuclease III footprinting data are an indication that EhPCNA stabilizes the ΔPIP complex similar to full‐length DNA ligase.

The DNA footprinting boundaries of *Chlorella* virus DNA ligase shows protection for eight or nine nucleotides at the 3′OH and 11–12 nucleotides at the 5′PO_4_ side and the crystal structure of human DNA ligase I in complex with nicked DNA predicts a protection at least 10 bp at the 3′OH of the nick, and 9 bp at the 5′PO_4_ side. Contrary to the expected based on the crystal structure of human DNA ligase I, the observed footprint for EhDNAligI comprises a total of 37 nucleotides instead of 20 nucleotides. However, it is important to mention that the crystal structure of the human enzyme lacks the first N‐terminal 262 amino acids and *Chlorella* virus DNA ligase only contains 298 amino acids in comparison to 686 amino acids of EhDNAligI. The exonuclease footprinting assay demonstrates that the specific binding between EhPCNA and EhDNAligI is not dependent of the PIP box and that the overall architecture of the ternary complex between full‐length and ΔPIP is similar.

### The IDCL and C‐terminal domain of EhPCNA are necessary to stimulate the enzymatic activity of EhDNAligI

**Figure 6:**
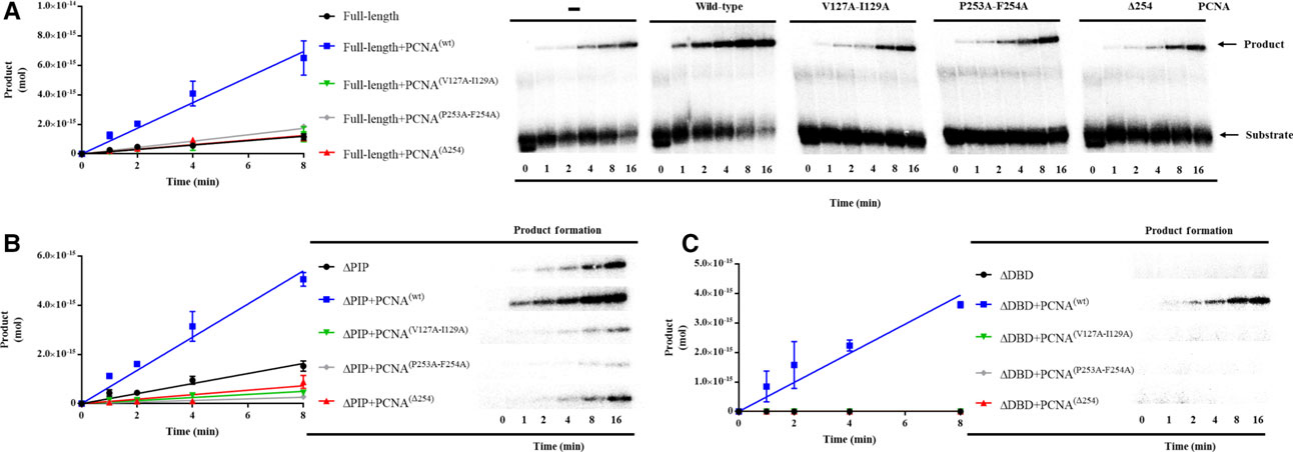
The IDCL and C‐terminal region of EhPCNA are needed for enzymatic activity stimulation of EhDNAligI. Reaction containing 500 fmol of [γ‐^32^P]‐labeled DNA substrate, 50 fmol of EhDNAligI (A), ΔPIP (B), or ΔDBD (C) in the presence of 150 fmol of wild‐type EhPCNA, an IDCL‐binding‐deficient mutant (EhPCNA‐V127A‐I129A), and two C‐terminal interacting region (EhPCNA‐Δ254 and EhPCNA‐P253A‐F254A). Reactions were incubated at room temperature for 0, 1, 2, 4, 6, and 8 min, aliquots were quenched at the indicated times adding stop buffer and loaded into a 15% polyacrylamide/8M urea gels. Data are presented as pmol of ligated product as function of incubation time.

In a seminal work, Vijayakumar and coworkers demonstrated that the C terminus of yeast PCNA is necessary to establish a functional interaction with yeast DNA ligase I 35. In this work, it was suggested that the initial interactions of yeast PCNA–yeast DNA ligase I occurs via PIP box; however, further contacts with the PCNA ring and DNA ligase I promote the ability of DNA ligase to encircle DNA 35. Structural data indicate the nature of these interactions: the PIP box of yeast DNA ligase I forms hydrophobic interactions with the C‐terminal region of PCNA (residues 251–255) and the IDCL (residues 121–132), specifically with a hydrophobic pocket mediated by residues Leu126 and Ile128. These data indicate that both the IDCL and C‐terminal interactions of PCNA would be necessary to stimulate nick‐sealing activity by DNA ligase I. In order to study the role of the C‐terminal region of PCNA to form a PCNA–DNA ligase complex, we constructed a series of EhPCNA mutants that disrupt IDCL binding (EhPCNA‐V127A‐I129A) and the C‐terminal interacting regions (EhPCNA‐Δ254 and EhPCNA‐P253A‐F254A) and test their effect on nick‐sealing stimulation of EhDNAligI (Fig. 6). C‐terminal EhPCNA deletion and point mutants and a mutant that compromises IDCL binding were unable to stimulate the activity of full‐length and ΔPIP EhDNA ligases I (Fig. 6A,B). Our results support the notion that IDCL and C‐terminal interactions of PCNA are necessary to establish a functional PCNA–DNA ligase I complex. In order to corroborate these results, we assayed the effect of the PCNA mutants in the ∆DBD mutant. As expected, all EhPCNA mutants were unable to stimulate ligase nick‐sealing activity for the ∆DBD ligase mutant (Fig. 6C). Our data are congruent with a previous study that indicate that mutations in the PIP box of yeast DNA ligase have a less substantial effect on the yeast PCNA–yeast DNA ligase interaction with respect to the mutation in yeast IDCL that drastically decrease the interaction.

### The activity of an EhDNAlig deficient in PIP box is stimulated by an intact EhPCNA but not by EhPCNA with mutations in the IDCL or C‐terminal domain

**Figure 7:**
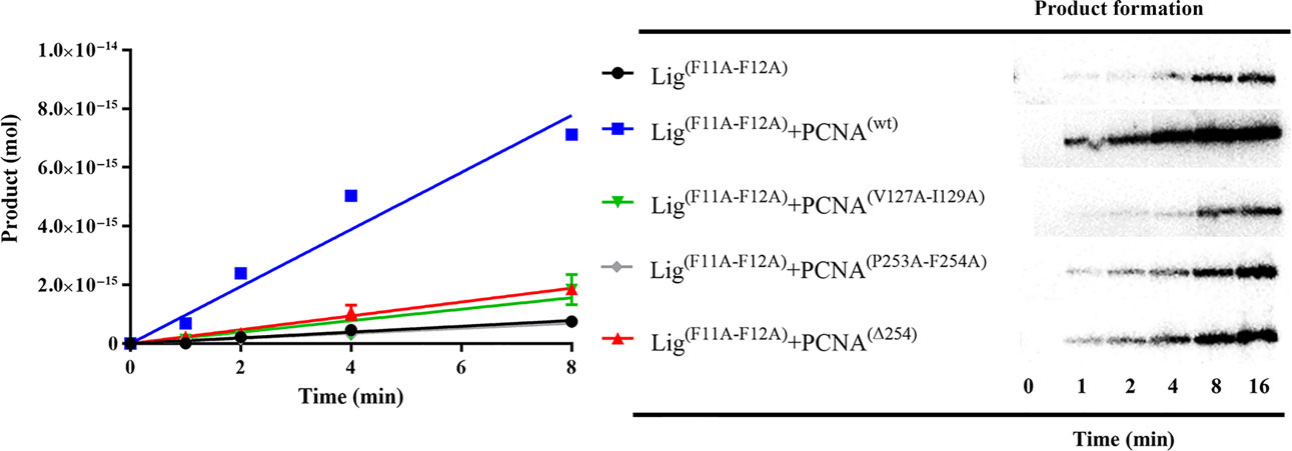
The activity of a PIP box mutant EhDNAlig is stimulated by wild‐type EhPCNA but not by EhPCNA‐harboring mutations in the IDCL or C‐terminal domain. Time course nick‐sealing reaction showing the amount of ligated product by a double EhDNAlig I‐Phel1Ala:Phe12Ala mutant in the presence of wild‐type and mutant PCNAs.

Because of the lack of stimulation of EhDNAlig by EhPCNA mutants, we were curious to test if EhDNAlig mutants that replace the most prominent hydrophobic interactions of the PIP box in EhDNAligI with EhPCNA would have an effect on nick‐sealing activity upon incubation with wild‐type and mutant forms of EhPCNA. We constructed a double EhDNAligI‐Phe11Ala:Phe12Ala mutant aimed to eliminate the aromatic residues of the PIP box 3_10_ α‐helix without completely eliminating its secondary structure 36, 37. We found that the enzymatic activity of EhDNAligI‐Phe11Ala:Phe12Ala mutant is simulated 9.8‐fold by wild‐type EhPCNA, supporting our previous results that the PIP box interaction is not necessary for nick‐sealing stimulation. However, EhPCNA mutants that compromise the binding to the EhPCNA′s IDCL (EhPCNA‐V127A‐I129A) and a C‐terminal EhPCNA deletion (EhPCNA‐Δ254) only stimulated nick‐sealing activity by < 2.4‐fold (Fig. 7).

## Discussion

PCNA stimulates the enzymatic activities of various enzymes that contain the PIP box interacting motif like Ape2, XPF, and FEN1 21, 38, 39. All these proteins share canonical PIP box sequences that promote their interaction with PCNA. However, the stimulatory effect of PCNA to the nick‐sealing of DNA ligases is controversial. Several reports established that PCNA is not involved in a rate enhancement of the nick‐sealing reaction by DNA ligase; however, other reports have shown a rate enhancement upon PCNA incubation 16, 21. In this work, we established that in the absence of a PIP box‐interacting motif, PCNA stimulates the nick‐sealing activity of DNA ligase I, and that PCNA is able to restore the catalytic activity of a truncated DNA ligase I, that by itself is unable to bind to the substrate, and therefore carry out the last step of the ligation reaction. An excess of PCNA increases the nick‐sealing activity by threefold for full‐length and ΔPIP EhDNA ligases. Interestingly, PCNA restores the enzymatic activity of a DNA ligase with impaired DNA binding capabilities. PCNA may stimulate the nick‐sealing activity of full‐length and ΔPIP and restore the activity of ΔDBD by increasing the affinity of the ligase to the nicked DNA substrate or by favoring a proper alignment of the catalytic amino acids residues with DNA by distorting DNA 16, 21, 39.

The PIP box sequence is highly conserved between DNA ligases, for example a substitution of a conserved Phe in the PIP box of *Aeropyrum pernix* DNA ligase results in a protein that is defective in PCNA binding 40. Likewise, a double mutant in the PIP box of *S. sulfataricus* (*Ss*) leads to a DNA ligase that is impaired in its ability to be stimulated by SsPCNA 16. *Entamoeba histolytica* and *H. sapiens* DNA ligases I contain canonical PIP boxes (Fig. 1A) that bind to the IDCL of their respective PCNAs. Many studies suggest the importance of the PIP box in PCNA‐mediated interactions 6, 41, 42; however, our knowledge regarding specific surface interaction between PCNA and its protein partners is still incomplete. Herein, we demonstrate that in the case of DNA ligase, the PCNA interaction is partially independent of the PIP box. Our kinetic and footprinting data indicate that the physical interaction between DNA ligase I and PCNA is partially independent of PIP box and that a conserved interface is needed to stimulate catalysis. Footprinting experiments of EhDNAligI shows a greater protection in the 3′OH than in the 5′PO_4_, this asymmetric protection pattern agrees with structural data of human DNA ligase I and previous footprinting patterns of other DNA ligases (Fig. 5). We found that wild‐type and ΔPIP EhDNAligI produced a clear footprinting pattern in the presence of EhPCNA, indicating that PCNA helps to stabilize the protein complex on nicked DNA. However, footprinting studies indicate that ΔDBD does not form a stable complex on nicked DNA even in the presence of EhPCNA. These data contrast to fluorescence anisotropy experiments in which the ΔDBD mutant in the presence of EhPCNA binds to nicked DNA with a *K*_d_ of 95 nm (Fig. 3). Furthermore, the absence of a stable footprinting for the ΔDBD–EhPCNA complex or EhPCNA alone contrasts with structural work that indicates that bacterial β‐clamp binds dsDNA with a *K*_d_ of 120 nm 43. It is possible that PCNA exhibits a decreased binding affinity for dsDNA in comparison to its structurally homolog β‐clamp, as yeast PCNA binds to dsDNA with a 100 μm affinity 44 or that our failure to observe a binding between ΔDBD and EhPCNA by footprinting maybe due to displacement of the protein complex by exonuclease III. Although this contradictory result, the appearance of nick‐sealing activity for ΔDBD in the presence of EhPCNA, in comparison to an inactive enzyme indicates that EhPCNA reorganizes the binding and catalytic activities of this ligase.

Our results using mutated C‐terminal and the mutated IDCL version of EhPCNA indicate that the PIP box of DNA ligase is not necessary for binding to PCNA. However, the C‐terminal of PCNA is necessary to stimulate DNA ligase catalysis. Crystal structure of yeast PCNA with a peptide derived from the PIP box of yeast DNA ligase shows that upon DNA ligase binding, the C terminus of PCNA becomes ordered and forms a β‐zipper structure 35. These structural data show that a DNA ligase–PCNA interaction that is not mediated by the PIP box is sufficient to induce a conformational change in PCNA 35. Mutations in the subunit interface of yeast PCNA compromise DNA repair responses by altering the Rad52‐mediated homologous recombination and the REV3 repair pathways 45. Interestingly, in the case of the Rad52 pathway, the subunit interface yeast PCNA mutants do not disrupt the binding to Rad54, suggesting that the regulatory effects of PCNA are not only mediated by binding to its target proteins. In light of this evidence, we suggest that the C‐terminal of EhPCNA may function also as an allosteric modulator of EhDNA ligase. Examples of allosteric modulation by PCNA occur in DNA polymerase δ 46 and protein kinase ATM 47. Recent work by the Aharoni laboratory indicates that PCNA interactions are not optimized for high binding affinity to their protein partners 48, and that the binding of PCNA to different proteins is mediated by coevolution 49. The notion that PCNA contains a variety of binding sites 45 and biochemical experiments that indicate that the ring‐shaped surface of PCNA binds to the DBD of human DNA ligase I facilitating the formation of a DBD ring‐shaped structure 17 is in agreement with the suggestion that PCNA may be involved in promoting an allosteric transition in EhDNAlig. We suggest that the interacting surfaces of EhDNAligI–EhPCNA complex are playing a role in the specificity of the interaction and activity stimulation. This putative interaction involving the surface of PCNA may occur in other protein complexes as the catalytic domain of human DNA ligase interacts with PCNA and the 9‐1‐1 complex in the absence of a PIP box motif 17.

**Figure 8:**
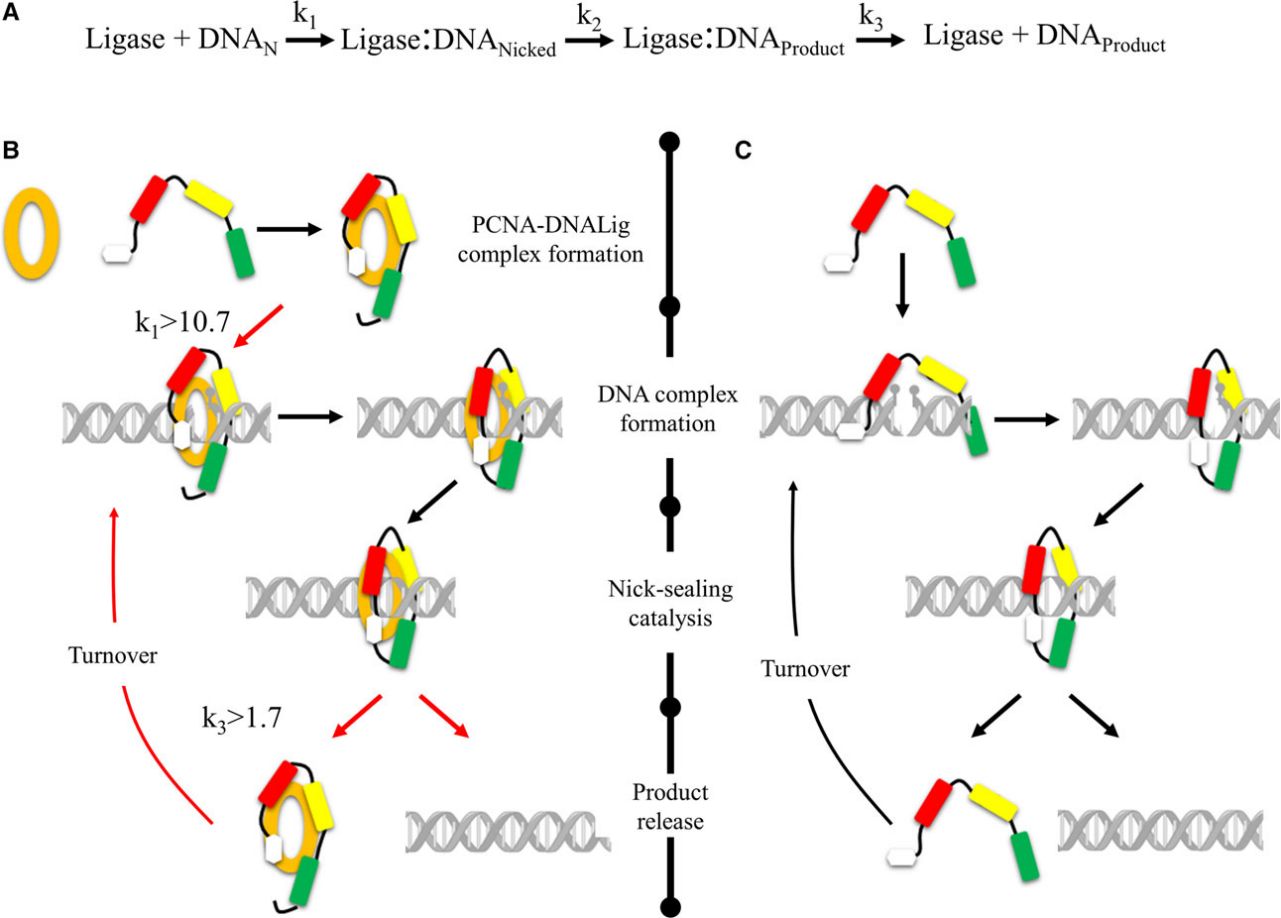
Schematic representation of the catalytic mechanism of DNA ligase and its structural changes involved in the presence and absence of PCNA. (A) Schematic representation of the three basic steps for nick‐sealing by EhDNAligI: DNA binding and nick identification, nick‐sealing catalysis, and product release whose rates are depicted by *k*_1_, *k*_2_, and *k*_3_, respectively. (B, C) Different structural changes involve in DNA ligase I in the presence and absence of PCNA. PCNA is showed in orange full‐closed ring, DNA ligase I is colored in the N‐terminal PIP box with white, DBD motif with red, adenylation in yellow, and Oligonucleotide‐oligosaccharide‐binding domain in green. The catalytic stages that are affected by influence of PCNA interaction are highlighted with red arrows indicating the increase.

The nick‐sealing mechanism of DNA ligase I can be simplified by the scheme depicted in Fig. 8A. Firstly, DNA ligase I binds to DNA and identify the nicked site (depicted by *k*_1_), followed by nick‐sealing activity (*k*_2_), then the sealed DNA is released to restart the catalytic process again (*k*_3_). Accordingly, to our biochemical data, the affinity of DNA ligase for nicked DNA (*K*_m_) increases, whereas the *k*_st_ values remain without significant change in the absence and presence of PCNA, respectively). This indicates that the catalytic events that are affected by PCNA binding to DNAligI are the binding of the nicked DNA and turnover event. Taken all together, the dispensability of PIP box for PCNA interaction and the observed conformational change in the yeast PCNA–DNAlig complex, we think that our data support a similar mechanism to the one proposed by Ellenberger and Tomkinson′s groups 9, 15, 16, 35 (Fig 8B). In this mechanism, in the absence of PCNA, DNAligI has to carry large structural changes previous to the nick‐sealing reaction followed by product release associated with return to the initial structural stage. However, the interaction of PCNA with DNA ligase promotes the formation of a pseudo ring‐shaped structure increasing the affinity for the nicked DNA followed by a fully ring‐shaped and catalytically active form. The catalytic rate (*k*_2_) is not affected by PCNA–DNAlig complex formation. Nonetheless, the *k*_3_ turnover is increased during the interaction favoring the product release without large structural changes.

## Materials and methods

### Construction and purification of EhDNAligI and EhPCNA mutants

EhDNAligI and EhPCNA mutants were generated from a pCold I‐EhDNAligI plasmid and a modified pET19b‐EhPCNA plasmid, respectively. Mutant proteins were purified as previously described 4, 25. The plasmid overexpressing HsPCNA was a gift from J. Pascal.

### Adenylation

Full‐length and deletion mutants of EhDNAligI were completely deadenylated as previously described 25. To determine the adenylation of EhDNAligI and deletion mutants, reactions of 20 μL with 1 μg of previously deadenylated proteins were incubated with 1 μCi [α‐^32^P]ATP for 10 min at room temperature in adenylation buffer [50 mm Tris/HCl (pH 7.5), 100 mm NaCl, 5 mm dithiothreitol (DTT), and 10 mm MgCl_2_]. The reactions were quenched by adding an equal volume of 2× SDS buffer and separated by 10% SDS/PAGE and visualized by phosphorimagery.

### DNA binding affinity measurement

The DNA binding affinity of EhDNAligI and deletion mutants was determined by measuring changes in the anisotropy of a nicked DNA substrate that was 3′‐FAM‐labeled. Reactions of 100 μL with 26 pmol of 3′‐FAM‐labeled nicked DNA were incubated for 2 min at 30 °C with increasing concentrations of full‐length and deletion mutants (0.39–100 pmol) in binding buffer (50 mm Tris/HCl (pH 7.5), 25 mm NaCl, 5 mm DTT, 10 mm MgCl_2_, 250 μg/mL BSA, and 1 mm ATP). The change in anisotropy was measured using a fluorescence intensity detector (TECAN Infinite M1000) with excitation and emission wavelengths of 495 and 520 nm, respectively. The *K*_d_ was estimated by fitting the data to the nonlinear equation for one site‐specific binding Y = (B max × X/(*K*_d_ + X)) + background). X corresponds to the molar concentration of EhDNAligI or EhDNAligI–EhPCNA complex, Y corresponds to the change in anisotropy and B max is maximum fluorescence change. All experiments were performed in triplicate.

### Antibodies and EhPCNA pull‐down

Rabbit polyclonal antibodies against EhPCNA were raised against purified protein 4. Purified (His)_6_‐tagged EhDNAligI and deletion mutants were incubated with EhPCNA in pull‐down binding buffer (50 mm Tris/HCl (pH 7.5), 100 mm NaCl, 5 mm DTT, and 10 mm MgCl_2_) at room temperature for 1 h with and without excess of DNA substrate_._ Nicked DNA contained a 3′ dideoxy nucleotide to avoid sealing. The samples were passed trough out a His‐Trap affinity column and washed with 50 volumes of binding buffer and eluted with binding buffer supplemented with 500 mm imidazol. Samples were run on a SDS/PAGE gel and transferred onto a polyvinylidene fluoride membrane. The blots were incubated with anti‐EhPCNA antibody at 1 : 9000 dilution and detected with horseradish peroxidase‐coupled secondary antibodies through ECL.

### Nick‐sealing template assembly

Two DNA substrates were generated as previously described 25. The first of them is a nicked double‐stranded DNA substrate in which a 45 mer template is annealed to 24 mer upstream and 21 mer downstream oligonucleotides. The upstream 24 mer (5′cgcagcccacctgcc cacctaact3′) contained a 3′OH and the downstream 21 mer (5′ggccctgcgctagtgccaagg3′) a 5′PO_4_. The second substrate was generated using a nicked DNA hairpin (5′ccttggcactagcgcagggccagttaggtgggcaggtgggctgcgttttcgcagcccacctgcccacctaact3′) with a downstream 21 mer similar as the first substrate. The 21 mer of both substrates were 5′PO_4_ radiolabeled with 100 μCi [γ‐^32^P] ATP and T4 kinase.

### DNA ligation assays

The ligation reactions were performed with the indicated amounts of substrate, full‐length, deletion mutants, EhPCNA, or HsPCNA. The standard reaction buffer consists of 50 mm Tris/HCl (pH 7.5), 1 mm ATP, 10 mm MgCl_2_, and 5 mm DTT. Reactions were incubated accordingly to the figure legends. Reaction were quenched by adding an equal volume of stop buffer (90% formamide (v/v), 50 mm EDTA, and bromophenol blue) and heated to 95 °C for 5 min. Reaction were run on 15% polyacrylamide/8M urea gels. Ligation products were detected by autoradiography and densitometry was performed on the scanned image using phosphorimagery. Three independents experiments were performed and the average of the standard deviation is indicated.

### Kinetic characterization under steady‐state conditions

Steady‐state kinetics were assayed in a reaction mixture of 20 μL with 500 fmol of substrate was incubated with 5 fmol of previously deadenylated full‐length and deletion mutants. The reactions were incubated for 16 min at room temperature and initiated by the addition of increasing ATP concentrations from 3.125 to 800 nm. The reactions were performed at room temperature to ensure that only the 10–15% of the substrate was converted to product. The *K*_m_, *k*_cat,_ and *V*_max_ values were determined by fitting experimental data to a Michaelis–Menten equation.

### Determination of rate constants in single‐turnover conditions

Determination of enzymes with functional active sites was performed under single‐turnover conditions in standard buffer in the absence of ATP. Reactions of 10 μL with 26 fmol DNA were incubated with increased concentrations of EhDNAligI and deletion mutants, from 3.6 to 116 pmol for 10 min at 37 °C. The relative percent of ligation was plotted as a function of enzyme concentration. The determination of the rate constant for single‐turnover ligation (*k*_st_) by full‐length and deletion mutants was performed with enzyme in excess respect to substrate and lacked exogenous ATP. Reaction mixture of 20 μL containing standard buffer free of ATP, with 52 fmol nicked DNA substrate. The reaction was initiated by the addition of enzyme at increasing concentrations from 40 to 320 fmol. The reaction was incubated at room temperature at the indicated times and quenched immediately. The relative percent of ligation was determined by phosphorimagery, calculating the percent of product. The time course reaction was fitted by nonlinear regression to the following equation 33:

$$F\left(t\right)=F_{\mathrm{ep}}\left(1-e^{K_{\mathrm{obs}}t}\right)$$

A double reciprocal plot of 1/*k*_obs_ versus 1/[enzyme] was fit to a straight line. The rate constant for single‐turnover ligation was determined from the *y*‐intercept (*y*‐intercept = 1/*k*_lig_) 50. The kinetics rates are measured in the course of a single enzyme aliquot to ensure consistency and minimize reduction in enzyme activity.

### Exonuclease footprinting

For DNA footprint studies, it was necessary the preparation of 2′,3′‐dideoxy substrate in the upstream position. First, a 23 mer strand (5′attcgcggcggtgctgatgcgtddC3′) with a dideoxy cytidine in the 3′OH position was annealed with a complementary 35 mer stand (5′attcgcggcggtgctgat gcgtcgtcggatgatt3′) and a downstream 12 mer (5′gtcggact gatt3′). Two different substrates were generated, the first labeled in the complementary 35 mer template and second in the upstream 12 mer, both radiolabeled with [γ–^32^P]ATP in the 5′PO_4_. A series of increased concentrations of EhDNAligI and ΔPIP (1–16 fmol) were incubated in binding buffer (50 mm Tris/HCl (pH 7.5), 6.6 mm MgCl_2_, 5 mm DTT) with 1 fmol of [γ–^32^P]‐labeled DNA substrate and increased concentrations of EhPCNA (3–48 fmol) where indicated. The samples were incubated at 4 °C for 15 min prior the addition of 15 U of exonuclease III and incubated at 20 °C for 8 min. The reactions were quenched by the addition of stop buffer and loaded onto a 15% polyacrylamide/8M urea gels.

## Author contributions

LGB, CHTA, EAL, and CSCF contributed to the experimental design. CHTA, EAL, CSCF, and CDQ performed the experiments. LGB, CHTA, CSCF, and EAL wrote the manuscript.

## Supporting information


**Fig. S1.** Amino acid sequence alignment of human and *E. histolytica* DNA ligase I proteins.

**Fig. S2.** Effect of HsPCNA on DNA binding capability of full‐length and deletion mutants.

**Fig. S3.** ∆DBD is unable to stimulate nick‐sealing.
