# Supplementary figures and images for: Proliferating cell nuclear antigen restores the enzymatic activity of a DNA ligase I deficient in DNA binding
Source: FEBS Open Bio. 2017 Mar 16;7(5):659–74. doi: 10.1002/2211-5463.12209 (PMC5407892; doi:10.1002/2211-5463.12209)

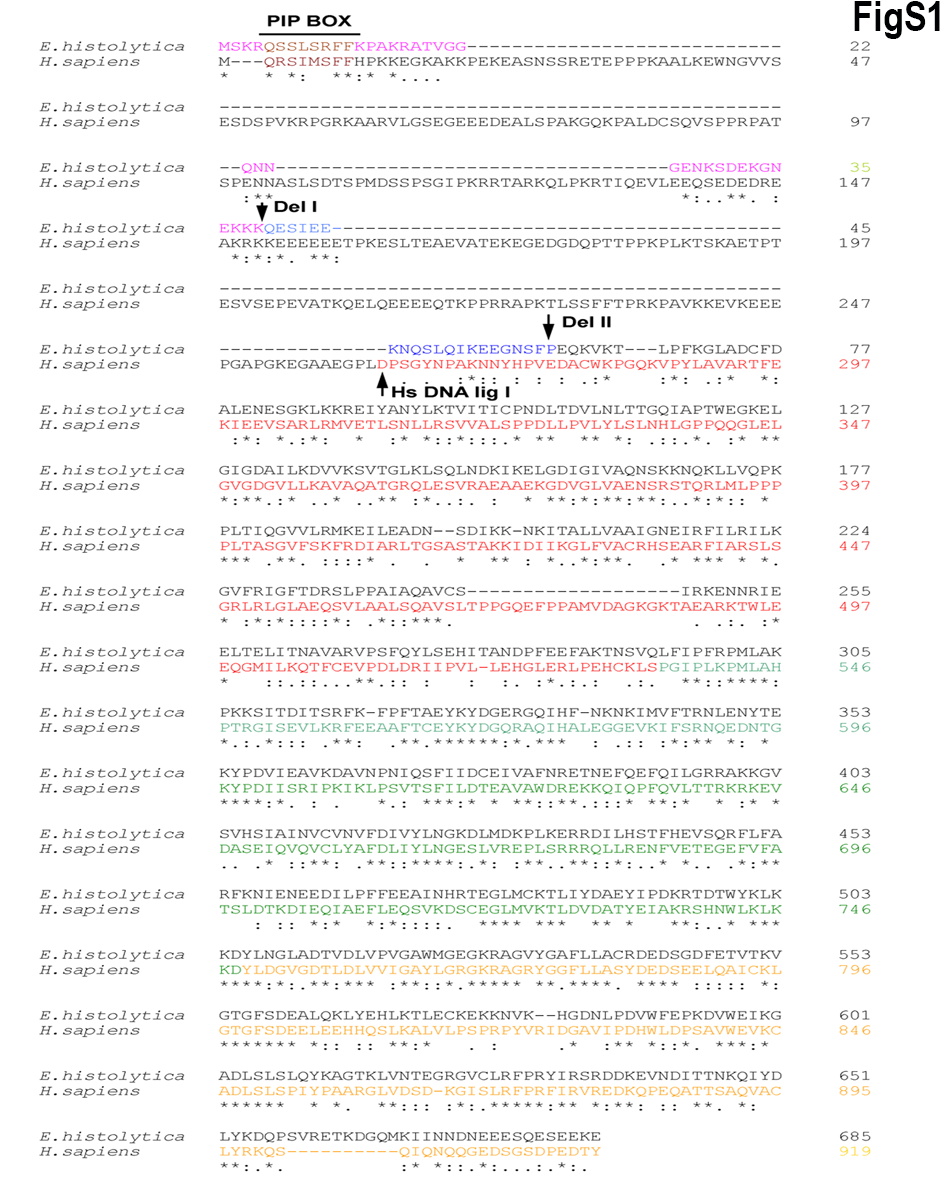

Supplement: Supplementary file 1 — Fig. S1. Amino acid sequence alignment of human and E. histolytica DNA ligase I proteins. [file FEB4-7-659-s001.tif]

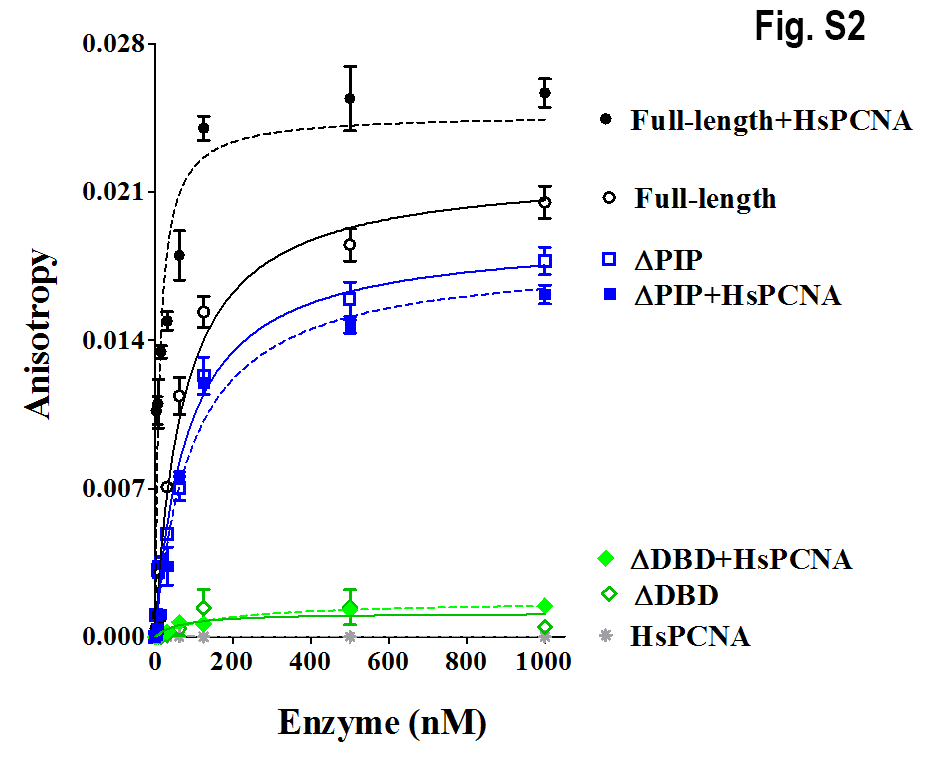

Supplement: Supplementary file 2 — Fig. S2. Effect of HsPCNA on DNA binding capability of full‐length and deletion mutants. [file FEB4-7-659-s002.tif]

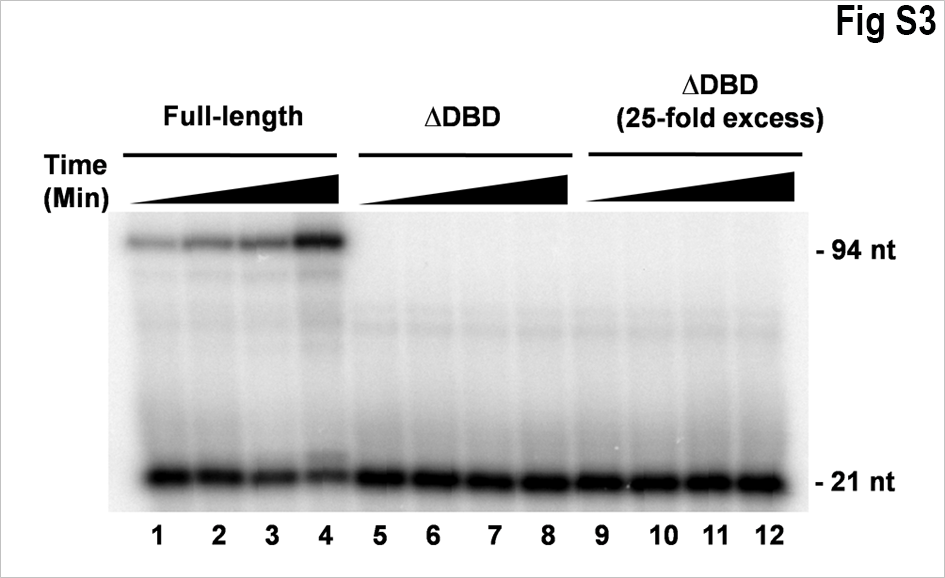

Supplement: Supplementary file 3 — Fig. S3. ∆DBD is unable to stimulate nick‐sealing. [file FEB4-7-659-s003.tif]
